# Co-expression network analysis of environmental canalization in the ascidian *Ciona*

**DOI:** 10.1186/s12862-022-02006-9

**Published:** 2022-04-28

**Authors:** Atsuko Sato, Gina M. Oba, Nathanael Aubert-Kato, Kei Yura, John Bishop

**Affiliations:** 1 Department of Biology, Ochanomizu University, Tokyo, Japan; 2 The Laboratory, Marine Biological Association of the UK, Plymouth, UK; 3 Department of Information Sciences, Ochanomizu University, Otsuka, Bunkyo-ku, Tokyo, Japan; 4 Department of Life Science & Medical Bioscience, Graduate School of Advanced Science & Engineering, Waseda University, Tokyo, Japan; 5 Human Life Innovation Center, Ochanomizu University, Tokyo, Japan; 6 Graduate School of Life Sciences, Tohoku University, Sendai, Japan

**Keywords:** Developmental buffering, Ascidians, Reciprocal cross, Maternal RNA, Co-expression analysis

## Abstract

**Background:**

Canalization, or buffering, is defined as developmental stability in the face of genetic and/or environmental perturbations. Understanding how canalization works is important in predicting how species survive environmental change, as well as deciphering how development can be altered in the evolutionary process. However, how developmental gene expression is linked to buffering remains unclear. We addressed this by co-expression network analysis, comparing gene expression changes caused by heat stress during development at a whole-embryonic scale in reciprocal hybrid crosses of sibling species of the ascidian *Ciona* that are adapted to different thermal environments.

**Results:**

Since our previous work showed that developmental buffering in this group is maternally inherited, we first identified maternal developmental buffering genes (MDBGs) in which the expression level in embryos is both correlated to the level of environmental canalization and also differentially expressed depending on the species’ gender roles in hybrid crosses. We found only 15 MDBGs, all of which showed high correlation coefficient values for expression with a large number of other genes, and 14 of these belonged to a single co-expression module. We then calculated correlation coefficients of expression between MDBGs and transcription factors in the central nervous system (CNS) developmental gene network that had previously been identified experimentally. We found that, compared to the correlation coefficients between MDBGs, which had an average of 0.96, the MDBGs are loosely linked to the CNS developmental genes (average correlation coefficient 0.45). Further, we investigated the correlation of each developmental to MDBGs, showing that only four out of 62 CNS developmental genes showed correlation coefficient > 0.9, comparable to the values between MDBGs, and three of these four genes were signaling molecules: BMP2/4, Wnt7, and Delta-like.

**Conclusions:**

We show that the developmental pathway is not centrally located within the buffering network. We found that out of 62 genes in the developmental gene network, only four genes showed correlation coefficients as high as between MDBGs. We propose that loose links to MDBGs stabilize spatiotemporally dynamic development.

**Supplementary Information:**

The online version contains supplementary material available at 10.1186/s12862-022-02006-9.

## Background

Development of organisms is adjusted toward one definite end-result irrespective of minor variation in environmental or genetic conditions. However, the environment can ultimately act as a ‘switch’ which shifts the end-result by altering developmental paths, providing novel phenotypes in the evolutionary process [1]. Canalization, or buffering, was originally defined by Waddington to describe developmental stability in the face of variable environmental or genetic conditions. The idea of environmental canalization has been a matter of debate over the past half a century. In addition to chaperone proteins [2–7], various other molecules and potential mechanisms have been identified as relevant to environmental canalization (see reviews, [8–10]). Other studies suggested that canalization is a property of gene networks [11–13]. In silico computer simulation showed that extracellular cell signaling plays an important role in stabilizing complex morphogenesis [14]. However, how buffering that stabilizes development is linked to the spatiotemporally dynamic processes of development at a transcriptome level remains to be discovered.

To this end, we exploit populations of sibling species of the ascidian *Ciona* that show a prominent difference in levels of canalization under thermal environmental variation [6] and occupy different, but overlapping, thermal ranges [15–19]. These populations have generally been known as *C. intestinalis* type A and *C. intestinalis* type B [16], although morphological differences between them have been identified [6, 16, 20, 21], and led to the reclassification of type A as *C. robusta* by Brunetti et al. [21]. For clarity and continuity with the previous literature, we use the ‘type A’ and ‘type B’ nomenclature here. *Ciona* release gametes to undergo external fertilization in the surrounding water, and gametes can be obtained by dissection to carry out planned fertilizations in the laboratory with large numbers of eggs from a given individual. These two species are found in sympatry in some areas and can be hybridized by mixing gametes in seawater [22–24]. Hybridization of these sibling species produces broods with different, maternally-determined levels of canalization depending on the gender (gamete) role of the respective parents [6, 24], which provides an excellent opportunity to explore the molecular basis of environmental canalization. In particular, study of reciprocal hybrid crosses showed greater control of the canalization of development in heat-shocked hybrid embryos with mothers of type A [6]. Here we investigate this finding further by examining patterns of gene expression and to what extent gene expression that correlates with buffering level behaves similarly to the gene expression of a well-defined developmental pathway. We found that buffering molecules are tightly linked to a small number of signaling molecules in the developmental pathway.

## Results

### Identification of maternal developmental buffering genes (MDBGs)

**Fig. 1:**
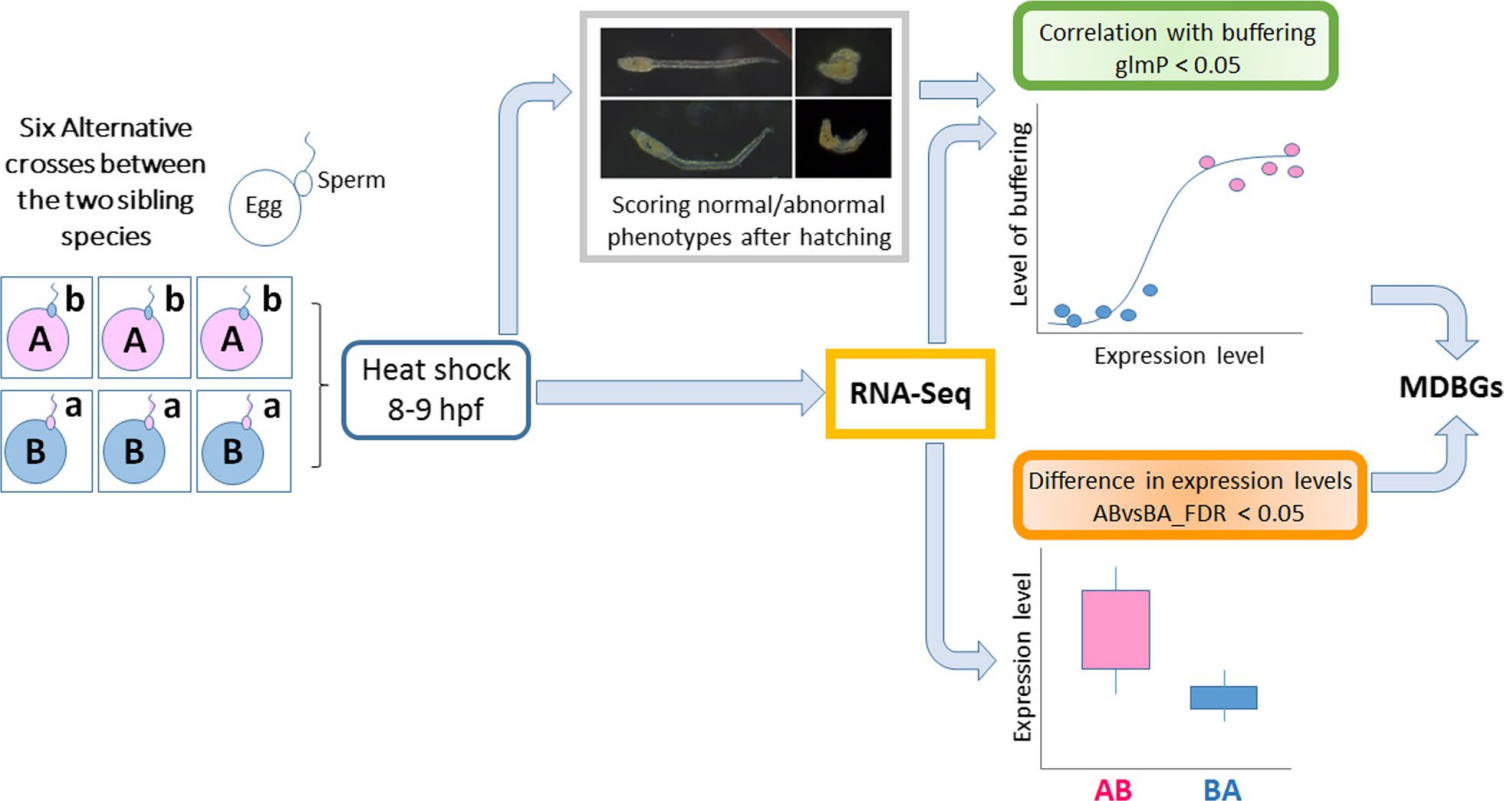
Experimental procedure. Type A and type B specimens collected from the wild were dissected to acquire gametes, and the species reciprocally crossed in vitro to generate three different hybrid broods for each alternative parental combination (i.e. six broods in all). At 8 h post fertilization (hpf), embryos were heat shocked at 27 °C for 1 h and a portion of each sample was collected for RNA-Seq. The rest of the embryos were then cultured at normal temperature from 9 hpf and the number of normally and abnormally developing larvae counted after hatching to measure the level of developmental buffering (Additional file 1: Table S3). After RNA-Seq, we conducted edgeR and generalized linear model analysis (glm) respectively to test gene expression level and developmental buffering level

Crosses of the sibling species type A and type B with the species in the alternative parental roles produce embryos with different developmental buffering levels [6]. Hybrid crosses (Additional file 1: Table S1) yielded three AB broods (type A mother) and three BA broods (type B mother), and were reared for 8 h at 17 °C, by which time they were at the neurula stage. Half of the embryos were exposed to 27 °C for 1 h, after which they had reached the early tailbud stage. Some of the embryos hatched abnormally, with a deformed notochord or the otolith and ocellus not properly formed, or even with whole-body deformation as shown in Sato et al. 2015 [6] (typical deformed phenotypes are shown in Fig. 1). The level of environmental canalization was significantly higher in AB samples than BA samples (Additional file 1: Fig. S1a, Table S3; *P* < 8.08 × 10^−16^), confirming a previous study showing this pattern of maternal control of environmental canalization [6].

Since previous studies showed that the developmental buffering level is maternally inherited, if a gene is involved in the maternal control of developmental buffering, we expect its expression level to be (i) positively correlated to the level of buffering, and also (ii) different between types AB and BA. We call a gene a ‘maternal developmental buffering gene’ (MDBG) if it meets both criteria. To assess (i), we first scored the proportion of normally developing heat-shocked progeny after hatching, as a measure of developmental buffering level (Fig. 1, Additional file 1: Table S3). Statistical analysis using a generalized linear model with quasibinomial errors showed that the expression levels of 458 genes out of 17,074 in total are significantly positively correlated to developmental buffering levels (glmP < 0.05; Additional file 1: Fig. S1b). (Here we call the *P*-value of this analysis glmP, to avoid confusion with *P*-values from other analyses.) These genes thus satisfied criterion (i).

To identify genes that meet criterion (ii), we compared the transcriptomes from AB embryos and BA embryos using edgeR [25]. We found 853 gene models that are differentially expressed between heated AB and BA embryos in the entire transcriptome (Additional file 1: Fig. S1d). These include genes showing negative correlation to the buffering level. However, since these genes may be involved in necrotic reaction in less canalized condition and difficult to distinguish from those involved in canalization, we analysed only these gene expressions positively correlated to buffering level. Amongst these, we found only 15 qualifying gene models (False Discovery Rate, FDR < 0.05) out of the 458 genes showing positive correlation to the buffering level; and thus we considered these to be MDBGs (Additional file 1: Fig. S1c,d; Additional file 2: Table S4). A previous study of yeast reported that the gene ontology terms (GO) of genes involved in environmental canalization were concerned with cellular homeostasis, such as DNA maintenance and organization, cell cycle, response to stimuli, RNA elongation and protein modification [26] rather than development as such. Supporting this, the 15 MDBGs include genes involved in the cell cycle (KY.Chr2.1529, Leucine aminopeptidase 3 (LAP3)), translation, stress response (KY.Chr2.2295, catalase), degradation in homeostasis (KY.Chr6.691, glycosylated lysosomal membrane protein (GLMP)), innate immunity (KY.Chr14.407; interferon regulatory factor 2 (IRF2)) and metabolism (Chr2.1046; galactocerebrosidase; KY.Chr9.550, speedy protein; Chr7.688, serine protease 27 (PRSS27)), while we did not find any transcription factors that are involved in development (Additional file 2: Table S4). We note that one of the other MDBGs, DnaJC10, was previously shown to be involved in canalization [6, 7].

To further understand maternal control of MDBGs, we undertook allele imbalance analysis using the embryonic transcriptome data (Additional file 2: Table S4). We found that, out of nine MDBGs that we analysed, six were maternally imbalanced, suggesting that MDBGs are mostly maternal transcripts.

### Identification of coexpression modules in environmental canalization

We next focused on understanding of the properties of MDBGs. Previous studies have uncovered properties of gene networks that cause canalization, such as having hub genes with high connectivity [26–28]. However, these studies were not performed at the transcriptome level. We aimed to investigate the property of buffering modules at the transcriptome level using our current data set. Since a protein–protein interaction network (PPI network) or other gene networks at the transcriptome level are not yet defined in *Ciona*, we identified coexpression modules correlated to environmental canalization using Weighted Gene Correlation Network Analysis (WGCNA) [29]. Using the data from 17,074 gene models in heat-treated embryos of the two hybrid genotypes (AB and BA), we identified 24 modules with WGCNA (Additional file 1: Figs. S2–S4). Notably, 14 out of 15 MDBGs were found in one of these modules, module 23 (Additional file 2: Table S4). The remaining MDBG was included in module 1. We named module 23 the ‘buffering module’. The buffering module consists of 3485 genes, the largest of all the modules identified (Additional file 1: Fig. S5a, b, Additional file 3: Table S5). The buffering module showed the lowest mean ABvsBA_FDR value overall and the third lowest mean glmP value (Additional file 1: Fig. S5c, d).

**Fig. 2:**
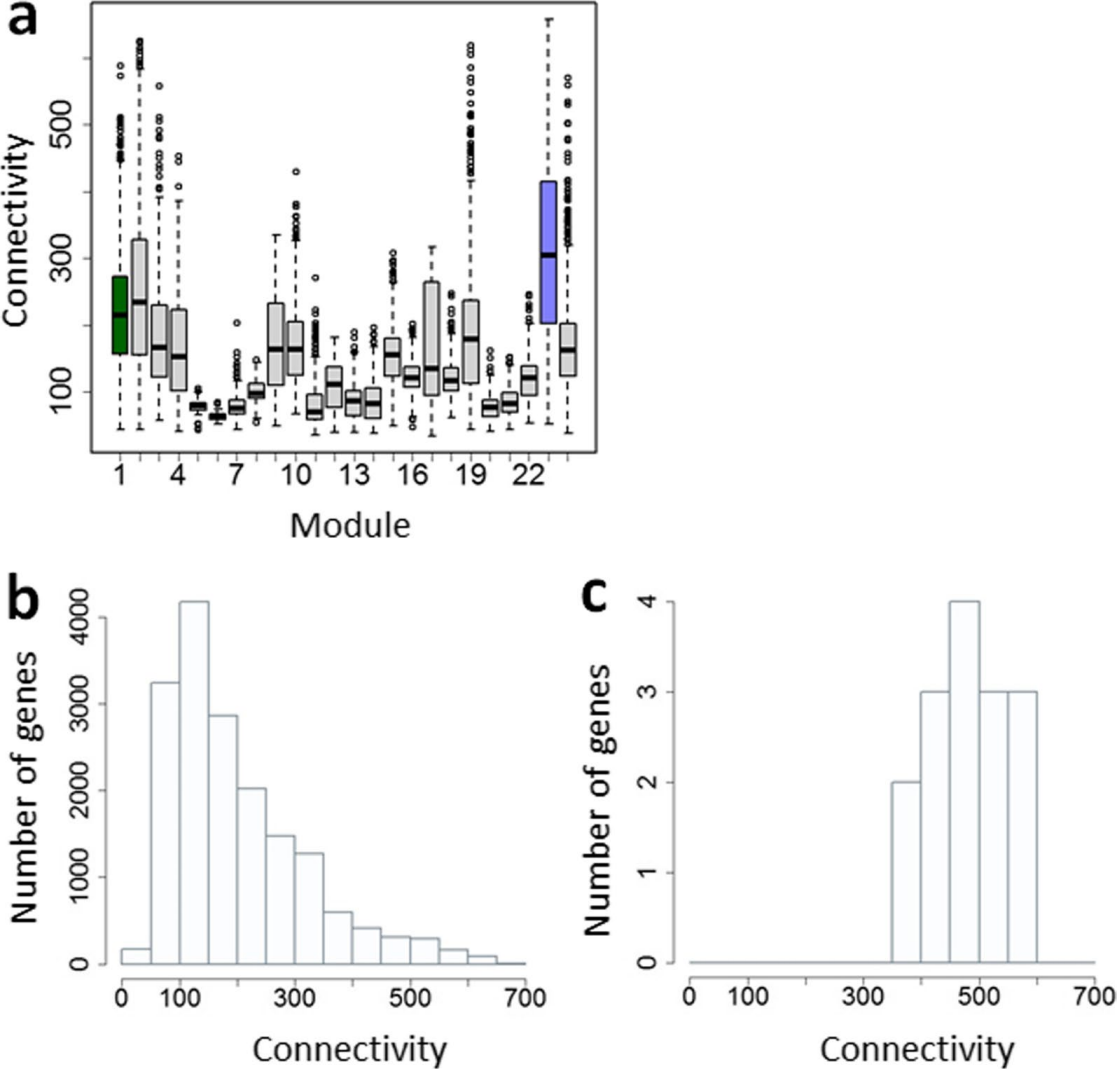
Connectivity coefficients of the coexpression modules and maternal developmental buffering genes (MDBGs). **a** Distribution of connectivity values of individual genes within each coexpression module across the transcriptome. Coexpression modules having MDBGs are colour-coded: Module 23 (14 MDBGs), blue; Module 1 (1 MDBG), dark green. **b** Frequency distribution of connectivity values of all genes in the transcriptome. **c** Frequency distribution of connectivity values of just the 15 MDBGs

To identify the characteristics of the buffering module and how MDBGs are integrated in the module, we calculated the connectivity of each gene to all the other genes in the transcriptome data and compared the distribution of those values in each module (Fig. 2a, Additional file 4: Table S6). The buffering module had the highest mean connectivity, which was significantly different from the rest of the modules (t-test, *P* < 2.2 × 10^−16^) (Fig. 2a). We also found that all the MDBGs have high connectivity (Fig. 2b, c; *P* < 9.068 × 10^−11^; mean connectivity of MDBGs was 477.7, whereas that of the whole network was 198.9). Since mean connectivity is significantly correlated to the module size (*F*-test, *P* < 2e−16; Additional file 1: Fig. S6a), we also compared connectivity of MDBGs within the buffering module to the connectivity of the other genes in that module. The mean connectivity of the MDBGs is significantly higher than that of the other genes in the buffering module (*t*-test, *P* < 1.44e−05; Additional file 1: Fig. S6b, c). Hence we confirmed that the module composed of genes with highest connectivity is correlated to environmental canalization, as shown in the previous studies on yeast [26–28], and that the MDBGs in the buffering module have even higher mean connectivity than the remaining genes in that module.

### Developmental pathway genes in the buffering module

**Fig. 3:**
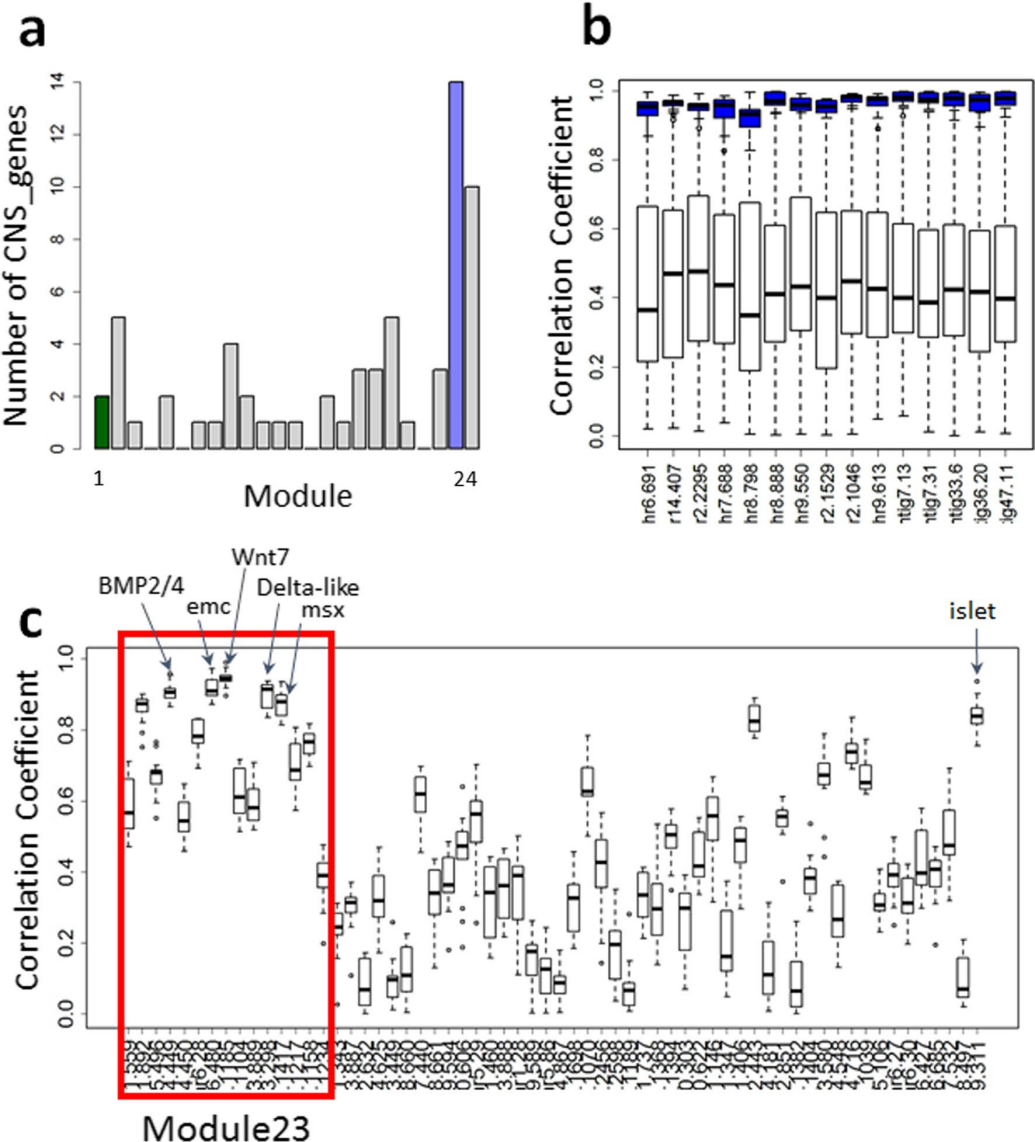
Genes identified in the gene regulatory network in *Ciona* central nervous system formation (‘CNS genes’) according to Imai et al. [30]. **a** Distribution of CNS genes in the 24 coexpression modules identified within the embryonic transcriptome. Note that CNS genes are present in 20 out of 24 modules but are well represented in the buffering module (Module 23), shown in blue; Module 1 is shown in green. **b** Correlation coefficients of all the CNS genes to each individual MDBG (white box-plots). Blue box-plots show correlation coefficients of each MDBG with the other MDBGs. All the values are shown in Additional file 8: Table S10 and Additional file 7: Table S9. **c** Correlation coefficient values of individual CNS genes to all the MDBGs (individual CNS-genes arrayed on *x*-axis). The CNS genes on the *x*-axis are in the same order shown in Additional file 7: Table S9; the CNS genes in the buffering module (Module 23) are indicated by the red box. Red line shows 0.9 in correlation coefficients. Red line shows 0.9 in correlation coefficients

The gene regulatory network studied by Imai et al. [30] is involved in the formation of the *Ciona* CNS, including the developmental stage at which our transcriptome data was collected. We found that the 62 CNS genes (Additional file 5: Table S7) were distributed across 20 out of the 24 modules (Fig. 3a, Additional file 6: Table S8). Fourteen of the 62 CNS genes (Fig. 3a) were found in the buffering module; this proportion did not differ significantly from the value expected by random distribution (*P* = 0.6377), given that the buffering module is a large module containing 3485 genes within the total transcriptome.

To understand how CNS genes are linked to MDBGs, we examined the absolute values of correlation coefficients between pairs of MDBGs, and between CNS genes and MDBGs. We found that MDBGs are more tightly connected to each other (Fig. 3b; average correlation coefficients is 0.96 ± 0.03) (Additional file 8: Table S10) than to CNS genes (where all the MDBGs show an average correlation of 0.45 ± 0.26) (Fig. 3c, Additional file 7: Table S9), showing that the developmental gene network is not centrally located in the buffering network, and only a small number of genes are tightly linked to MDBGs. To investigate which developmental genes are strongly linked to MDBGs, we also examined correlation coefficients between each CNS gene and MDBGs. Only one gene showed an average correlation coefficient > 0.93, comparable to the values between MDBGs: Chr8.1185 (Wnt7). Three other genes, Chr3.267 (*emc*), Chr 4.449 (BMP2/4), and Chr3.298 (Delta-like) showed correlation coefficients above 0.9 but lower than 0.93. Importantly, apart from Chr3.267 (*emc*), these genes are involved in signaling pathways.

## Discussion

We found 15 MDBGs that are both correlated to the developmental buffering level and show a marked maternal effect. Fourteen of these genes are included in a large co-expression module of high connectivity. While we used a relatively small sample size for WGCNA, our results confirmed previous studies on yeast and *Caenorhabditis elegans* showing high connectivity of genes in a canalized network [26–28, 31]. These MDBGs include DnaJC5, a chaperone previously shown to confer developmental buffering [6, 7] and five un-annotated genes that are located 3′ downstream of a gene encoding 28S rRNA. It is of note that these five genes encoding a part of 28S rRNA were all identical short sequences of only 215 bp, located downstream of 28S rRNA transcribed from different 45S rDNA loci. These sequences do not appear in the results of BLAST searches against other well-characterised genomes, such as mammals, fruit flies, and the worm *Caenorhabditis elegans*. Moreover, other rDNA genes did not show any correlation to the environmental buffering level, so we suggest that the short RNA sequence acts separately from rRNAs. Recent studies indicate the importance of small RNAs derived from 5′ upstream of 28RNA [32] or tRNA in stress responses and development [33, 34]. Further study is required to identify the role of short sequences from 45S rRNA in developmental and environmental canalization.

How spatiotemporal developmental pathways are integrated into canalization and how canalization relates to general homeostasis has been a matter of debate since Waddington clearly separated developmental buffering from general homeostasis [35]. Here we found that developmental pathways are integrated with environmental canalization via a small number of MDBGs and a small number of genes in the developmental network we studied. The observed loose integration of MDBGs and a developmental pathway might allow dynamic gene regulation of development through time and space. Our data also indicates that a small number of genes in a developmental pathway that are linked to MDBGs are responsible for determining the developmental pathway and therefore evolutionary innovation under changing environmental temperatures. A recent in silico study showed that cell signaling stabilizes development against noise [14]. Supporting this idea, our data also showed that three of the top four developmental genes showing an average correlation coefficient > 0.9 with the MDBGs were signaling molecules. How expression of these extracellular signaling molecules tightly-linked to MDBGs are destabilized under thermal stress and whether the response in gene expression under thermal stress differs between lineages of *Ciona* would be interesting future questions to address. Our data has several limitations. For example, our data was generated using whole embryos, not at a single-cell level. Furthermore, our data is limited to RNA, and no other materials, while studies of metabolites have shown that cellular metabolic state is tightly coupled with development [36]. Testing our hypothesis in a larger dataset including the metabolome at single-cell resolution will be a future challenge.

A maternal effect on environmental canalization has also been reported in other aquatic organisms, such as sea urchins [37] and several fish species [38, 39]. Since embryonic development is particularly susceptible to thermal stress, further studies of the role of these maternally provided materials in controlling environmental canalization will contribute to understanding how development was modified by the environment in the past and will be affected in the future by anthropogenic influences including global warming.

## Conclusions

In this study, we identified 15 MDBGs that are both correlated to the developmental buffering level and show a marked maternal effect. They are tightly correlated to each other, showing an average correlation coefficient of 0.96 ± 0.03. On the other hand, an experimentally identified gene network involved in the formation of the *Ciona* CNS showed an average correlation coefficient of 0.45 ± 0.26 to MDBGs, suggesting that this developmental gene network is not centrally located within the buffering network. We found that out of 62 genes in the developmental gene network, only four genes showed correlation coefficients as high as between MDBGs. We propose that loose links to MDBGs stabilize spatiotemporally dynamic development.

## Materials and methods

### Animals, eggs and embryos

Adult *C. intestinalis* of types A (*C. robusta*) and B (*C. intestinalis*) were collected from either Queen Anne’s Battery or Sutton Harbour, adjacent sites in Plymouth, UK, during summers (July and August) in 2016–2017 and kept in tanks under identical conditions at 17 °C and continuous light for 1–2 days until dissection. Animals were fed a mixture of *Rhinomonas reticulata* and *Isochrysis galbana* once a day. The specimens were retrospectively confirmed as pure-bred individuals of type A or type B by genotyping their sperm as described by Sato et al. [20]. Eggs and sperm were collected and fertilized as described previously [20, 22] and used in hybrid crosses between different pairs of individuals of types A and B, as shown in Fig. 1. We refer to progeny of crosses using type A eggs and type B sperm as type AB, and to progeny of crosses using type B eggs and type A sperm as type BA. Heat-shock experiments were conducted as previously described by Sato et al. [6] (Fig. 1). In brief, broods of embryos at 8 h post fertilization (hpf) at 17 °C, by which time they were at the neurula stage, were split and either heat-shocked by transfer to an increased temperature (27 °C) for 1 h or (not shown in Fig. 1) maintained at the control temperature of 17 °C. We previously established this experimental protocol as the minimum heat shock to see the difference in phenotypic outcome between reciprocal hybridizations of the sibling species. At 8 hpf, all the chordate characteristics of the central nervous system start to appear, and the gene network investigated experimentally by Imai et al. [30] is relevant to this stage. In each brood a portion of heat-shocked embryos was collected for extracting RNAs, and both heat-shocked and control embryos were reared at 17 °C and the number of progeny developing normally was counted after hatching at 22 hpf. We define the developmental buffering level as the proportion of normal development after hatching in the heat-shocked embryos [6]. Only crosses in which > 84% of embryos developed normally in control conditions were used for further analysis.

For egg transcriptome analysis, adults of types A and B growing side by side in a marina were collected, dissected and eggs were obtained from the oviduct. These adults were different individuals from the parents used to produce embryos for embryonic transcriptome analysis.

### RNA isolation, purification and cDNA library construction

RNA from hybrid embryos was isolated and purified as previously described [6], with the exception that we used Isogen (Nippon Gene) rather than Trizol (Invitrogen), and sequenced using Illumina HiSeq by Hokkaido System Science. 21–23 million reads per sample were obtained and processed for analysis (Additional file 1: Table S1).

RNA from unfertilized eggs was isolated using Isogen (Nippon Gene), purified and any DNA contamination was removed using MagMax (Applied Biosystems) according to the manufacturers’ instructions. The RNA of eggs and embryos was isolated and purified on different dates using different methods. However, we confirmed that the quantity of RNA collected and purified per egg or embryo did not differ significantly (Additional file 1: Fig. S7; ANCOVA, *P* = 0.929). Quality of the purified RNA was checked using Bioanalyzer 2100 (Agilent) and only samples that showed an RNA Integrity Number (RIN) > 8 were used for cDNA library construction. cDNA libraries were constructed using NEBNext ultra directional RNA library prep kit for Illumina (NEB, England) according to the manufacturer’s instructions.

### Sequencing analysis

The workflow of sequence analysis is shown in Additional file 1: Fig. S8. The adaptor sequences were trimmed from all reads by Trimmomatic [40], and trimmed reads > 10 bp in length were kept. Trimmed paired reads were mapped onto the HT *Ciona intestinalis* type A genome [41] using robust mapping software, BWA-MEM [42] with the default settings, yielding over 90% of paired reads mapped onto the genome. We found about 1% more reads mapped onto the masked HT genome from crosses using type A eggs than from crosses using type B eggs (Additional file 1: Table S2). Since the *Ciona* genome is highly polymorphic, we masked the HT genome to adjust the mapping bias between the samples, using either the six RNA-Seq analyses from type B eggs or all three RNA-Seq analyses of the type BA tailbud embryos. To seek a better way of masking the genome, we examined the allelic imbalance of genes on the mitochondrial genome, which should be maternally expressed, using the Ensembl genome that includes the mitochondrial genome (https://asia.ensembl.org/). We found better results when using reads from all three BA embryo batches to mask the genome (Additional file 1: Fig. S9). Therefore, we used the genome masked by the three BA embryonic sequences in the analysis.

We employed Allele Workbench (AW) [43], a pipeline for analyzing allele-specific expressions, using the Variants.pl batch script to identify SNPs using all the reads with a quality above 10 without base alignment quality computation. Using the vcf file created by Variants.pl with parameter t = 01, which takes SNPs with biallelic expression, and collecting SNPs that were shared between at least five samples amongst the six transcriptome data sets, we generated a masked genome using GSmask.pl in AW. All reads were mapped again to the masked genome to minimize the difference in the proportion of type A-originated mapped reads versus that of type B-originated mapped reads. Mapped read counts were summarized by featureCounts [44] and differentially expressed genes were statistically analyzed by edgeR [25] using the read counts, treating the False Discovery Rate (FDR) < 0.05 as the cut-off for differential expression. Transcription factors were identified by searching for gene names in the gene annotation of the HT genome [41].

Correlation between the levels of buffering and gene expression was assessed with a generalized linear model using the quasibinomial function in R [45]. Transcripts per million (TPM) was calculated to obtain the normalized gene expression level (x_gene_) in three heat-treated samples of crosses using type A eggs and type B sperm (ABH) and three crosses of type B eggs and type A sperm (BAH). The developmental buffering level was measured by counting the number of progeny showing normal development (N_n_) and abnormal development (N_a_) after hatching in each sample. Therefore the model was:

$${\text{glm}}\left( {{\text{cbind}}\left( {{\text{N}}_{{\text{n}}} ,{\text{N}}_{{\text{a}}} } \right)\sim {\text{x}}_{{{\text{gene}}}} ,{\text{family}} = {\text{quasibinomial}}} \right)$$

and those correlations with *P* < 0.05 were identified as significant.

### Allelic imbalance

We used AW (Souderlund et al. [43]), with some modifications, to identify allelic imbalance (AI). The number of SNPs was counted using snpASE.pl in AW to generate bed files from the reads mapped to the masked HT genome. We created in-house python scripts (see footnote) to sort the bed files and extracted the number of SNPs the same as the reference type A genome (= Ref) and the same as the type B genome (= Alt), and calculated the ratio AI:

$${\text{AI}} = {{{\text{Ref}}} \mathord{\left/ {\vphantom {{{\text{Ref}}} {\left( {{\text{Ref}} + {\text{Alt}}} \right)}}} \right. \kern-\nulldelimiterspace} {\left( {{\text{Ref}} + {\text{Alt}}} \right)}}$$

for each gene. We then calculated the average of AI in type AB samples (AI_AB_) and in type BA samples (AI_BA_) and genes having |AI_AB_–AI_BA_|> 0.3 were defined as being in allelic imbalance according to Xu et al. [46].

### Network analysis

We identified coexpression modules correlated to environmental canalization using weighted correlation network analysis, WGCNA [29], following the tutorial. Ideally, we would undertake functional screening to identify gene networks. However, conducting functional genetic screening of thousands of genes was beyond our resources. To investigate the gene network here, we investigated and categorized genes by their patterns of gene expression based on the idea that genes in the same gene regulatory modules are expected to be co-expressed [47]. This method will not resolve causative or functional relationships between the genes. However, we believe that WGCNA, using the correlation pattern of gene expression, is an unbiased approach to extracting groups of genes of related function. In brief, we first normalised the transcriptome data to TPM, then computed the gene expression similarity s_*ij*_ of genes Gene_*i*_ and Gene_*j*_ in the transcriptome data as the absolute value of their correlation coefficient. We then computed an adjacency matrix A = (a_*ij*_) describing the connections between genes by applying a soft threshold β to the correlation matrix for all pairs *i,j* a_*ij*_ = s_*ij*_^β, which allows us to compute the connectivity of Gene_*i*_ as the sum of the values of all the pairs in the adjacency matrix that involved Gene_*i*_:

$${\text{Connectivity}}_{i} = \sum\nolimits_{i \ne j} {a_{ij} }$$

β is called a soft threshold and is used to keep information about the strength of correlations while minimizing the impact of weak correlations. While one could also use a hard threshold for this purpose (all values below the threshold are considered 0, others are kept as they are), doing so will give as much impact to numerous weaker connections as to a few strong connections. Setting β allows us to give a larger impact to strong connections, which are more relevant to the study of modules. The value of soft threshold β was determined by following the instructions of WGCNA [29]. In the current data, we found β = 18 for hybrid embryonic transcriptome data to be an appropriate threshold to identify modules (Additional file 1: Fig. S10).

### Developmental pathway genes in the buffering module

To examine to what extent developmental pathways are integrated with buffering, we calculated correlation coefficients between MDBGs and genes involved in an experimentally tested gene regulatory network characterized by Imai et al. [30]. These genes are involved in the formation of the *Ciona* central nervous system (CNS) including the developmental stage where our transcriptome data was collected. Here we call these genes ‘CNS genes’.

## Supplementary Information

**Additional file 1: Figure S1.** Identification of maternal developmental buffering genes (MDBGs) by comparing alternative hybrid crosses. **Figure S2.** Linkage hierarchical clustering dendrogram of the genes. **Figure S3.** Topological Overlap Matrix among all genes regarding genotype. **Figure S4.** Topological Overlap Matrix among all genes regarding robustness. **Figure S5.** Identification of buffering module by comparing alternative hybrid crosses. **Figure S6.** Connectivity of the buffering module. **Figure S7.** Comparisons of mRNA yields from different samples. **Figure S8.** Workflow of sequencing analysis. **Figure S9.** Allelic imbalance of mitochondrial genes using different parameter values in Variants.pl in AW. **Figure S10.** Determining the threshold value for WGCNA analysis. **Table S1.** Embryo transcriptome sample details, sequencing data obtained, and associated developmental buffering level. **Table S2.** Mapped reads to the HT genome in the transcriptome samples. **Table S3.** Developmental buffering data in each sample used for sequencing. H indicates heat stressed samples, and C indicates control sample.

**Additional file 2: Table S4.** Summary of MDBGs.

**Additional file 3: Table S5.** Module size.

**Additional file 4: Table S6.** Connectivity of each gene.

**Additional file 5: Table S7.** List of 62 CNS_genes used for the analysis.

**Additional file 6: Table S8.** Summary of number of CNS_genes in each module.

**Additional file 7: Table S9.** Correlation coefficient of between CNS_genes and MDBGs.

**Additional file 8: Table S10.** Correlation coefficient of between MDBGs.

## Data Availability

DNA sequences: Deposited to DDBJ Project Number PSUB014723. Code: binbucket.org/atsukos/maternal-control-of-canalization.

## Abbreviations

CNS: Central nervous system
GO: Gene ontology
hpf: Hours post fertilization
MDBG: Maternal developmental buffering gene, in which the expression level is positively correlated to the level of buffering, and also different between progeny types AB and BA
WGCNA: Weighted correlation network analysis
