# Supplementary material for: Co-expression network analysis of environmental canalization in the ascidian Ciona
Source: BMC Ecol Evol. 2022 Apr 28;22:53. doi: 10.1186/s12862-022-02006-9 (PMC9052645; doi:10.1186/s12862-022-02006-9)
Supplement: Supplementary file 1 — Additional file 1: Figure S1. Identification of maternal developmental buffering genes (MDBGs) by comparing alternative hybrid crosses. Figure S2. Linkage hierarchical clustering dendrogram of the genes. Figure S3. Topological Overlap Matrix among all genes regarding genotype. Figure S4. Topological Overlap Matrix among all genes regarding robustness. Figure S5. Identification of buffering module by comparing alternative hybrid crosses. Figure S6. Connectivity of the buffering module. Figure S7. Comparisons of mRNA yields from different samples. Figure S8. Workflow of sequencing analysis. Figure S9. Allelic imbalance of mitochondrial genes using different parameter values in Variants.pl in AW. Figure S10. Determining the threshold value for WGCNA analysis. Table S1. Embryo transcriptome sample details, sequencing data obtained, and associated developmental buffering level. Table S2. Mapped reads to the HT genome in the transcriptome samples. Table S3. Developmental buffering data in each sample used for sequencing. H indicates heat stressed samples, and C indicates control sample. [file 12862_2022_2006_MOESM1_ESM.pdf]

Supplementary Information

Co-expression network analysis of environmental canalization in the ascidian *Ciona*

Figure S1 Identification of maternal developmental buffering genes (MDBGs) by comparing alternative hybrid crosses.

Figure S2 Linkage hierarchical clustering dendrogram of the genes.

Figure S3 Topological Overlap Matrix among all genes regarding genotype.

Figure S4 Topological Overlap Matrix among all genes regarding robustness.

Figure S5 Identification of buffering module by comparing alternative hybrid crosses.

Figure S6 Connectivity of the buffering module.

Figure S7 Comparisons of mRNA yields from different samples.

Figure S8 Workflow of sequencing analysis.

Figure S9 Allelic imbalance of mitochondrial genes using different parameter values in Variants.pl in AW.

Figure S10 Determining the threshold value for WGCNA analysis.

Table S1 Embryo transcriptome sample details, sequencing data obtained, and associated developmental buffering level.

Table S2 Mapped reads to the HT genome in the transcriptome samples.

Table S3 Developmental buffering data in each sample used for sequencing.

Additional file 2: Table S4 Summary of MDBGs.

Additional file 3: Table S5 Module size.

Additional file 4: Table S6 Connectivity of each gene.

Additional file 5: Table S7 List of 62 CNS_genes used for the analysis.

Additional file 6: Table S8 Summary of number of CNS_genes in each module.

Additional file 7: Table S9 Correlation coefficient of between CNS_genes and MDBGs

Additional file 8: Table S10 Correlation coefficient of between MDBGs.

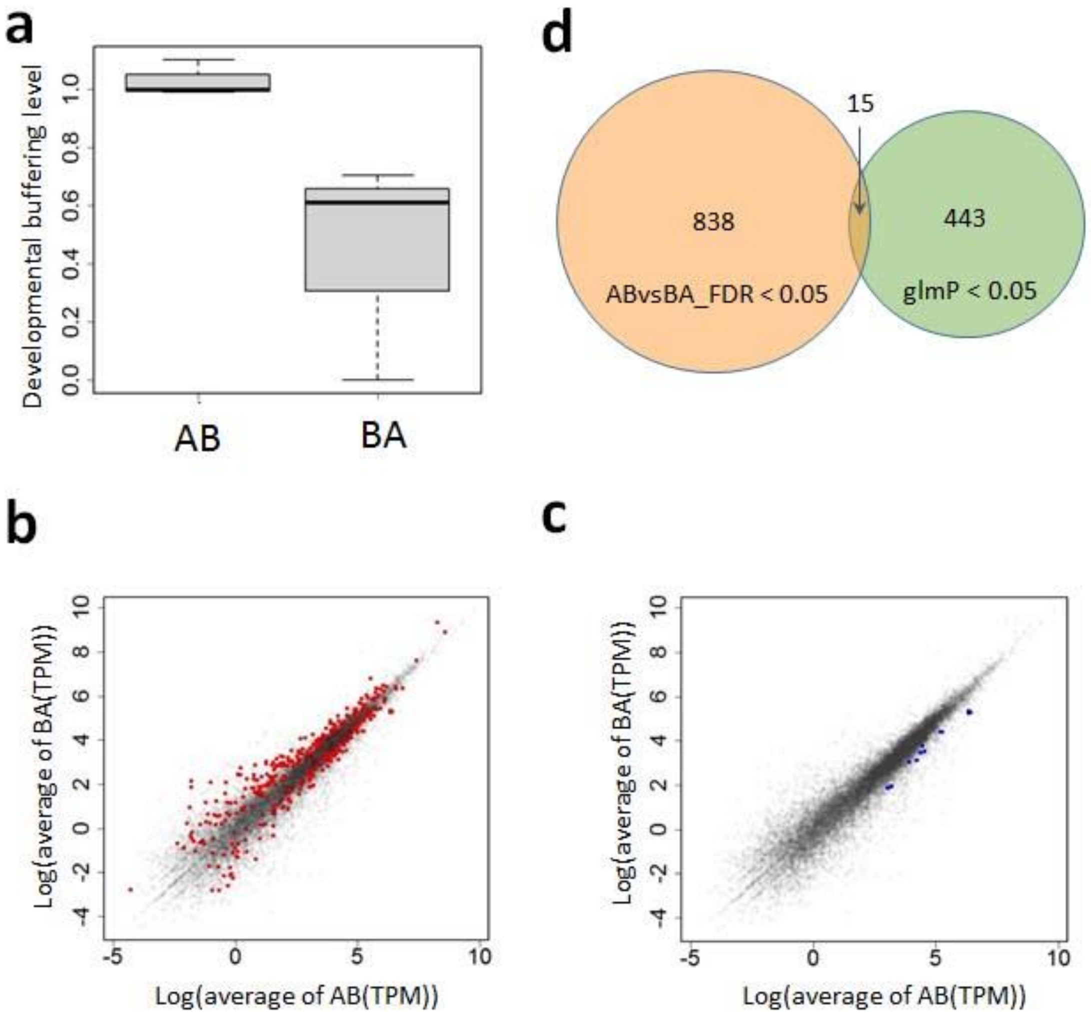

Figure S1 Identification of maternal developmental buffering genes (MDBGs) by comparing alternative hybrid crosses. (a) Difference in developmental buffering level between AB hybrids and BA hybrids used for RNA-Seq analysis, showing significant difference between the hybrids with opposite parental roles (P = 8.08e-16). (**b**) Transcriptome data of AB and BA showing 458 genes with glmP < 0.05 in red. **(c)** Transcriptome data of AB and BA showing 15 MDBGs in blue. **(d)** Venn diagram showing number of genes identified in edgeR and glm analysis, with 15 genes (MDBGs) in region of overlap.

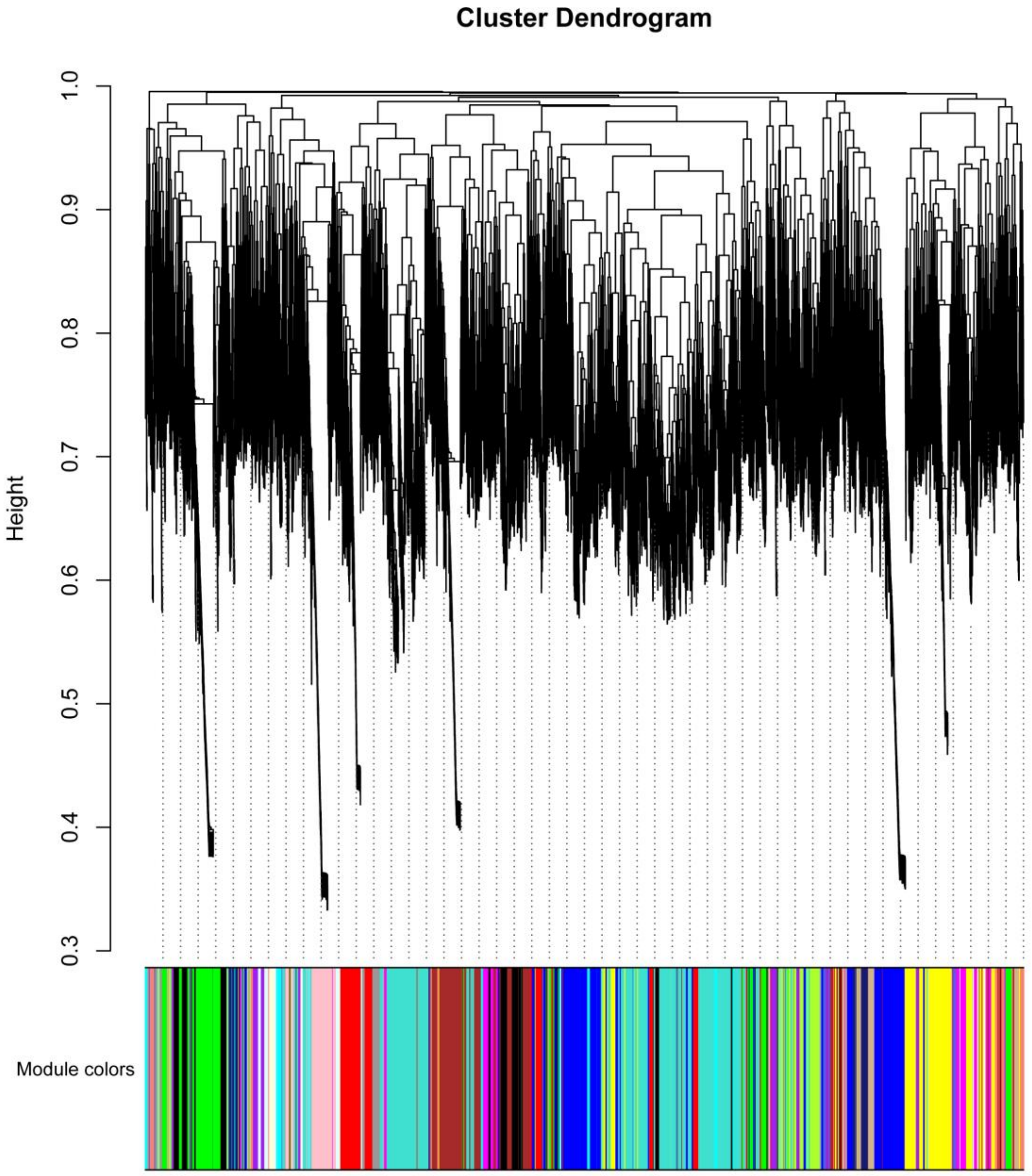

Figure S2 Linkage hierarchical clustering dendrogram of the genes. (a) Module 23 is shown in turquoise.

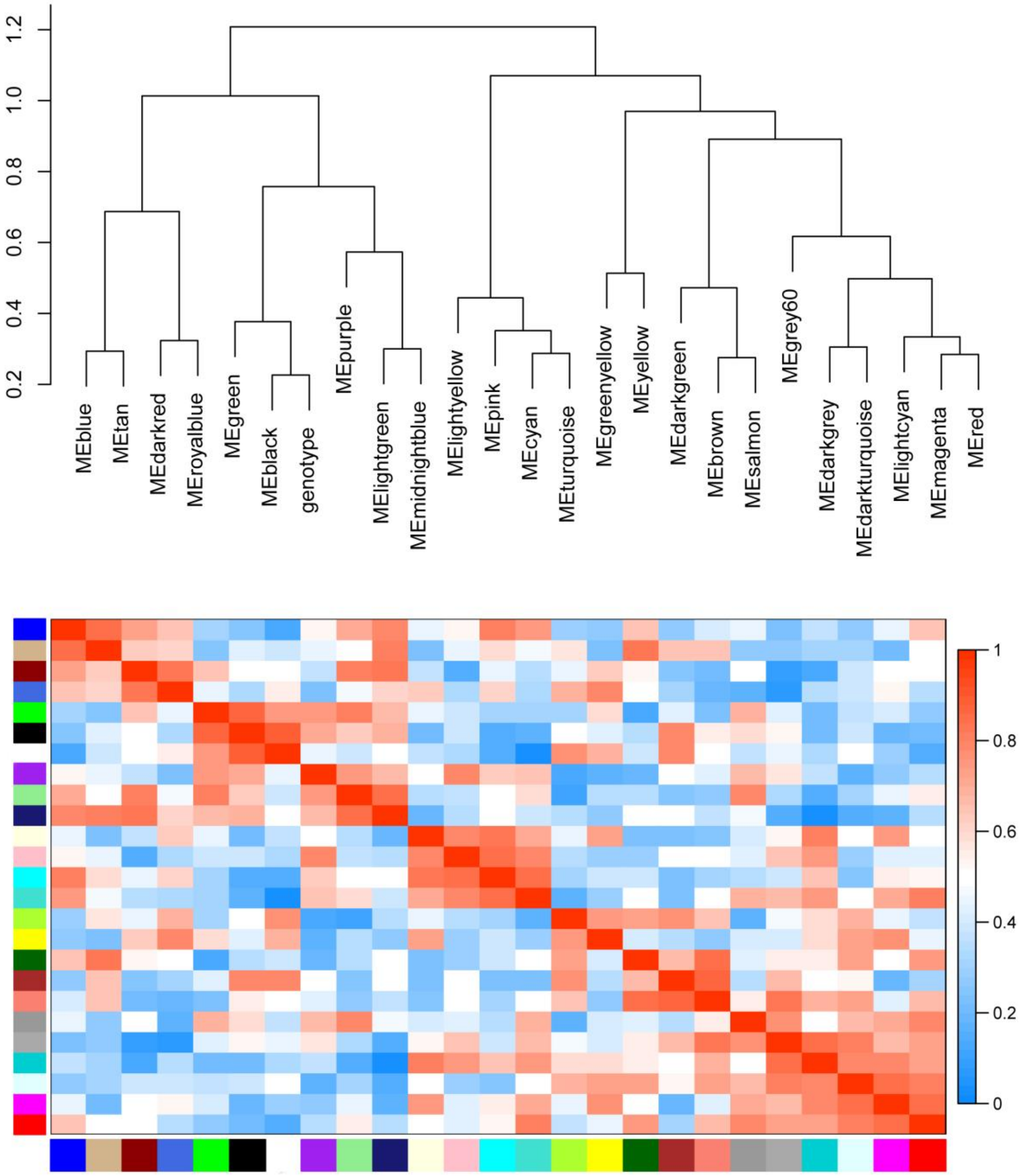

Figure S3 Topological Overlap Matrix among all genes regarding genotype. For colour code of each module, see Table S6. Genotype is shown in white, and the module 23 is shown by turquoise.

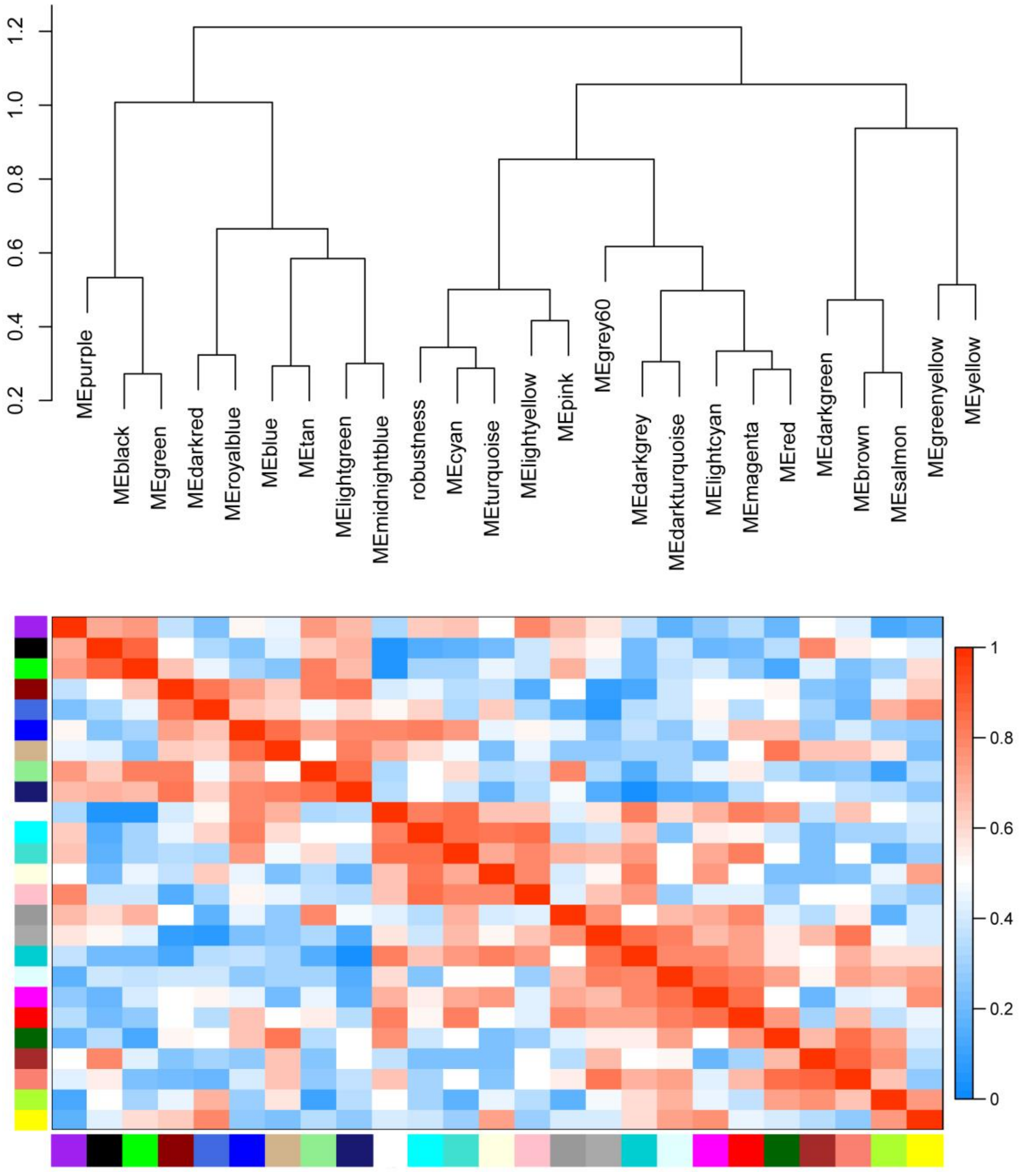

Figure S4 Topological Overlap Matrix among all genes regarding robustness. For colour code of each module, see Table S6. Robustness is shown in white, and the module 23 is shown by turquoise.

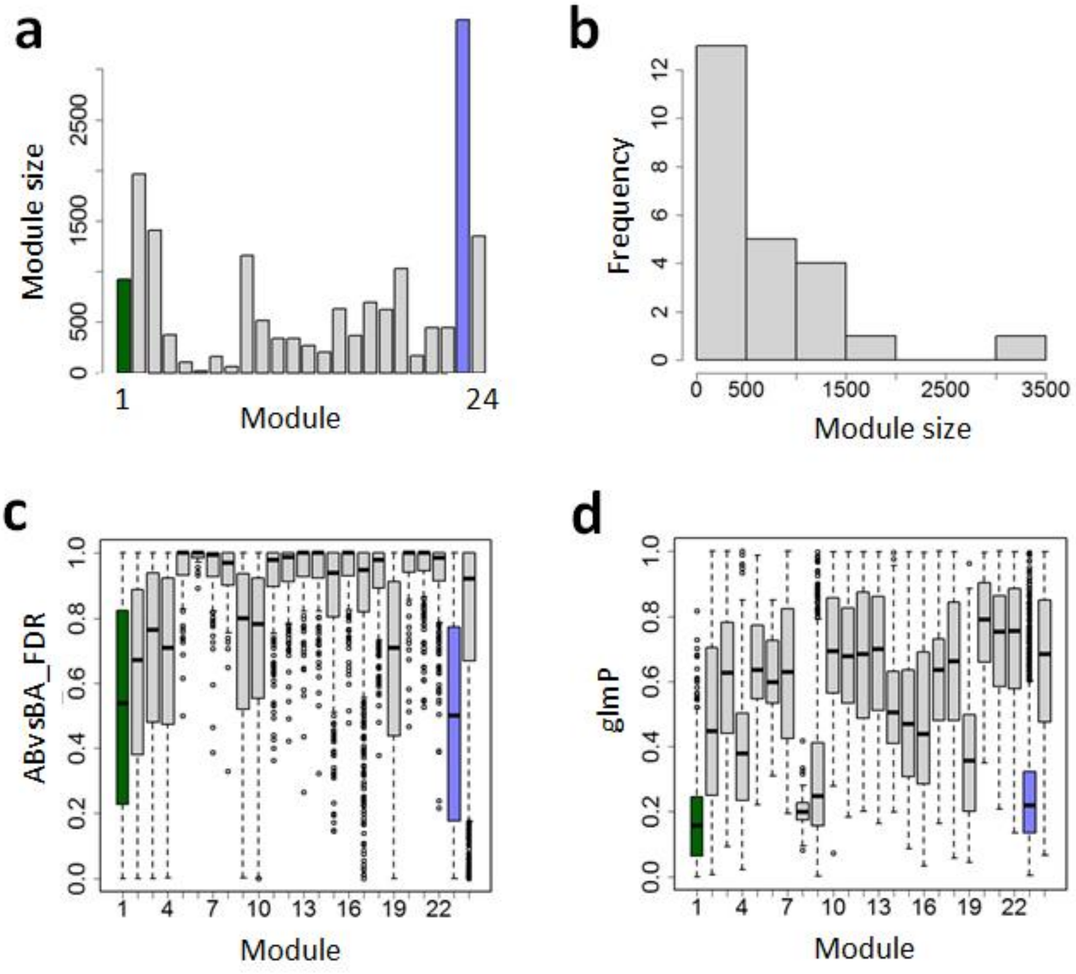

Figure S5 Identification of buffering module by comparing alternative hybrid crosses. (a) Number of genes (module size) of each gene co-expression module. (**b**) Distribution of module size in the entire network. **(c)** ABvsBA_FDR values of each module. **(d)** glmP values of each module. Colour code for **(a)**-**(d)**: Module 23 (the buffering module), blue; module 1, dark green.

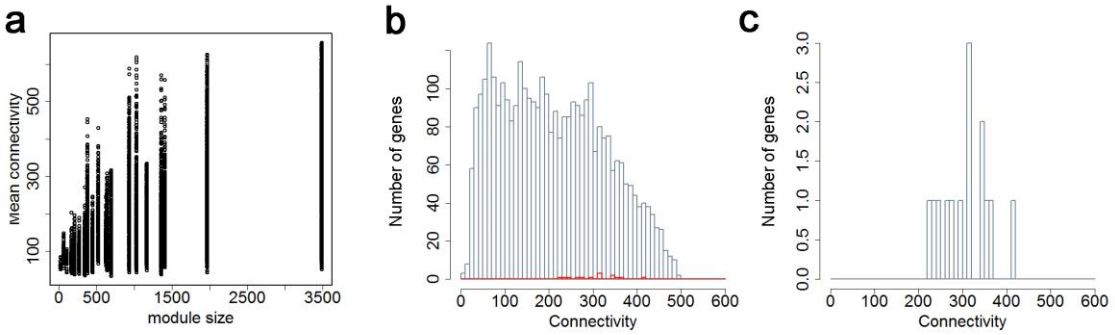

Figure S6 Connectivity of the buffering module. (a) Distribution of mean connectivity of each module according to the number of genes involved in each module. (**b**) Frequency of mean connectivity of genes in the buffering module. Histogram shown in red is the distribution of connectivity of the MDBGs in the module, which is magnified in **(c)**.

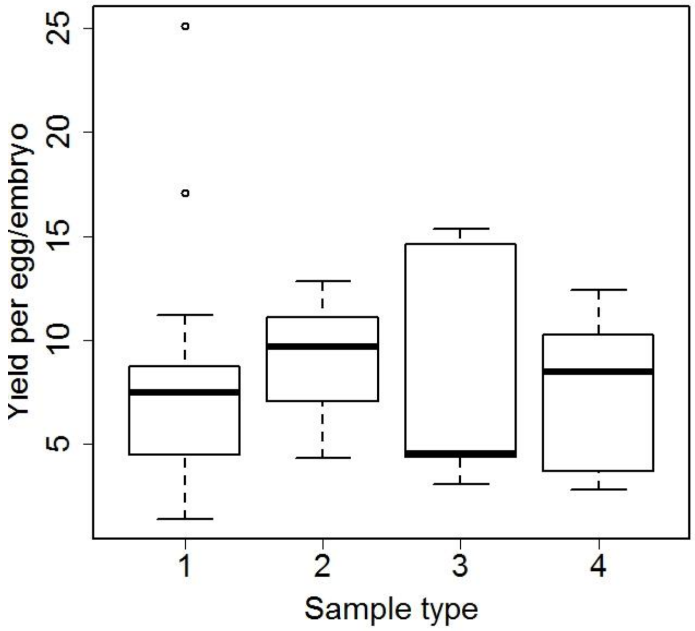

Figure S7 Comparisons of mRNA yields from different samples. Sample type 1, type A egg (n = 16) purified with MagMax (Applied Biosystems); sample type 2, type B egg (n=7) purified with MagMax (Applied Biosystems); sample type 3, hybrid embryos purified with MagMax (Applied Biosystems) (n=5); sample type 4; hybrid embryos purified with RNA micro Purification kit (Qiagen) and used for transcriptome analysis in this manuscript. (n=12). Note that RNA yields per egg or embryo did not differ significantly (P = 0.929).

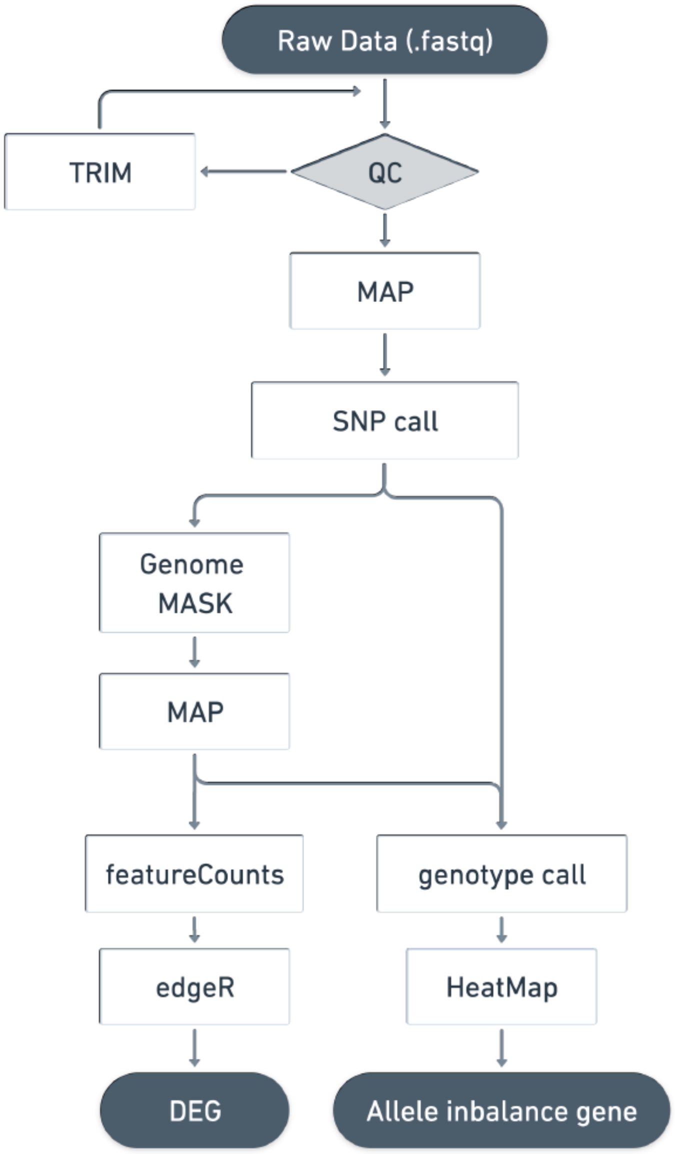

Figure S8 Workflow of sequencing analysis

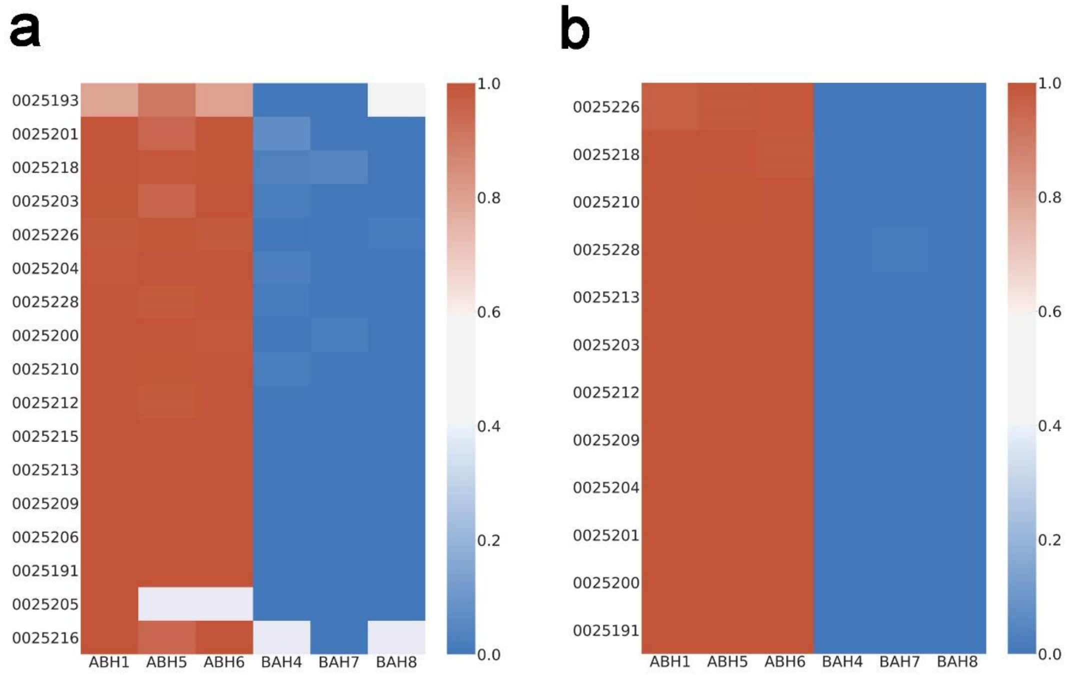

Figure S9 Allelic imbalance of mitochondrial genes using different parameter values in Variants.pl in AW. (**a**) t=11, m=5. (**b**) t= any, m=3. Note that the parameter values in (b) showed maternal expression of mitochondrial genes in both types A and B mitochondrial genomes, suggesting that the parameter values in (b) are appropriate for the imbalance analysis. Numbers on the y-axis indicate the last 7 digits of Ensemble gene ids, and broods are identified at the bottom (x-axis). AI values are colour coded; higher values show maternal expression in AB samples but paternal expression in BA samples, whereas lower values show paternal expression in AB samples but maternal expression in BA samples.

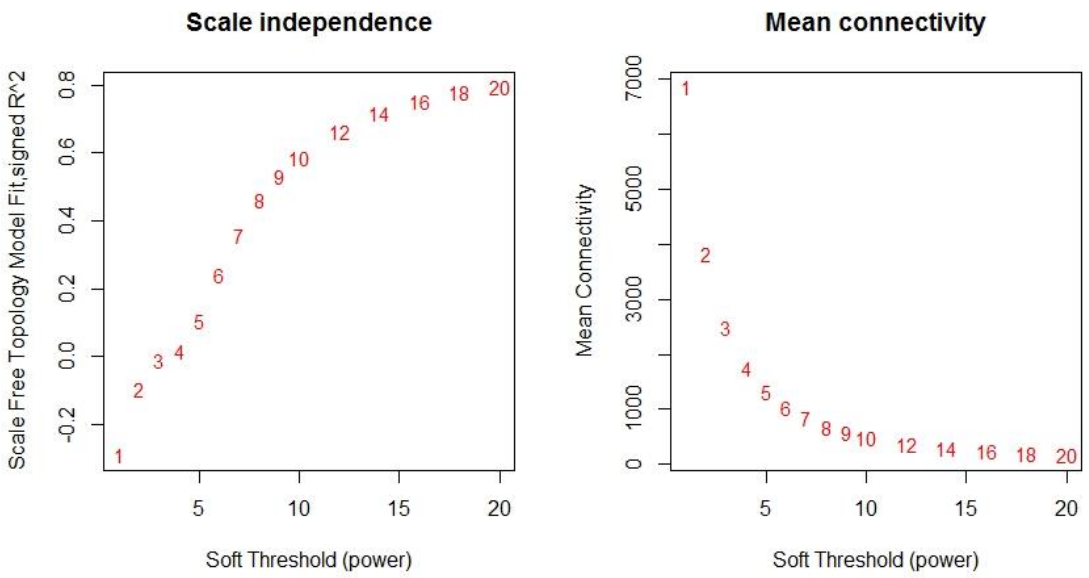

Figure S10 Determining the threshold value for WGCNA analysis. Plotted numbers show the values of β. We chose β=18, which gave a high fit value for the scale-free topology model combined with low mean connectivity

Table S1 Embryo transcriptome sample details, sequencing data obtained, and associated developmental buffering level.

Sample name	Egg donor ID	Sperm donor ID	Total reads	Developmental buffering level
**ABH1**	33	36	22,340,788	1.101609658
**ABH5**	9	12	22,100,888	0.995951417
**ABH6**	2	4	21,843,828	0.988888889
**BAH4**	36	33	21,592,656	0.610722611
**BAH7**	3	1	23,019,314	0
**BAH8**	24	22	21,355,594	0.638195882

Table S2 Mapped reads to the HT genome in the transcriptome samples.

	Data used to mask genomes	Parameters	ABH1	ABH5	ABH6	BAH4	BAH7	BAH8
Total reads	3BAH transcriptome	t=any, m=3	23522095	23179987	22499776	22062511	23920531	22002058
Total mapped reads			22884799	22551368	21846761	21363560	23138767	21273801
Paired reads			18636226	18369994	17496920	17584104	19282998	17513504
Paired			90.03%	89.81%	88.34%	89.10%	89.72%	88.79%
Total reads	RNA-Seq of type B eggs	t=11, m=5	23644689	23323220	22637486	22101933	23959103	22053450
Total mapped reads			23263416	22963667	22252854	21641165	23361100	21561494
Paired reads			19114034	18841852	17988156	18028772	19683212	17950196
Paired			92.34%	92.12%	90.82%	91.35%	91.58%	91.01%
Total reads	3BAH transcriptome	Unmasked	23801629	23485618	22783984	22100378	23932851	22026915
Total mapped reads			23801629	22841512	22113697	21336917	23082702	21234987
Paired reads			18614304	18353882	17478914	17529036	19221226	17458962
Paired			89.93%	89.73%	88.24%	88.82%	89.43%	88.51%

Table S3 Developmental buffering data in each sample used for sequencing. H indicates heat stressed samples, and C indicates control sample.

Sample_name	Mother ID	Father ID	Number of normal larvae	Number of abnormal larvae
ABH1	33	36	219	12
ABH5	9	12	246	1
ABH6	2	4	89	1
ABC1	33	36	142	23
ABC5	9	12	130	0
ABC6	2	4	156	0
BAH4	36	33	66	55
BAH7	3	1	0	101
BAH8	24	22	202	102
BAC4	36	33	117	14
BAC7	3	1	59	0
BAC8	24	22	137	8

Additional file 2: Table S4 Summary of MDBGs. E-val, pvalues of blast search showing level of homology to the annotation in the column B; PValue, p-value obtained from the edgeR analysis; AI, allele imbalance value, >3 shows maternal imbalance (see method); Module, module number by WGNCA analysis. Values of the column O-T shows TPM values of in each transcriptome sample for each gene. ABH_BAH shows differences of average TPM values between ABH and BAH (average TPM of ABH minus average TPM of BAH).

Additional file 3: Table S5 Module size.

Additional file 4: Table S6 Connectivity of each gene.

Additional file 5: Table S7 List of 62 CNS_genes used for the analysis.

Additional file 6: Table S8 Summary of number of CNS_genes in each module.

Additional file 7: Table S9 Correlation coefficient of between CNS_genes and MDBGs

Additional file 8: Table S10 Correlation coefficient of between MDBGs.
